# The Omega-3 Fatty Acids Eicosapentaenoic Acid and Docosahexaenoic Acid Enhance the Effects of Temozolomide Chemotherapy in Glioblastoma Cells

**DOI:** 10.3390/ijms26188759

**Published:** 2025-09-09

**Authors:** Janaína Alessandra Silva, Alison Colquhoun

**Affiliations:** Department of Cell and Developmental Biology, Institute of Biomedical Sciences, University of São Paulo, São Paulo 05508-000, Brazil; janaina.alessandra.silva@usp.br

**Keywords:** omega-3 fatty acids, glioblastoma, temozolomide, drug resistance, apoptosis

## Abstract

Overcoming resistance to temozolomide (TMZ) chemotherapy is a major challenge in glioma treatment. Polyunsaturated fatty acids (PUFAs) can interfere with drug resistance in glioma but their mechanism of action is poorly understood. Eicosapentaenoic acid (EPA) or docosahexaenoic acid (DHA) effects were assessed using proliferation and clonogenic assays, adhesion and migration assays, glucose and lactate metabolism, detection of lipid droplets, acidic vesicles, reactive oxygen species, and phosphokinase proteome analysis. EPA or DHA caused significant reductions in cell number, colony formation, adhesion, and migration in comparison with TMZ alone, increasing lipid droplet accumulation, reactive oxygen species formation, acidic vesicle number and apoptosis, while also altering glucose and lactate metabolism. TMZ increased phosphorylation of ERK 1/2, GSK3α/β, AMPKα1, Akt 1/2/3, PRAS40, CREB, HSP27, WNK1, and c-Jun in U87MG TMZR cells. EPA in the presence of TMZ reduced the phosphorylation of GSK3α/β, Akt 1/2/3, HSP27, and WNK1, while DHA reduced the phosphorylation of ERK 1/2, GSK3α/β, AMPKα1, Akt 1/2/3, and WNK1, thereby leading to additive or synergistic effects of EPA or DHA in combination with TMZ. Overall, the present study highlights the alterations seen in TMZ-resistant glioma cells when exposed to EPA or DHA and demonstrates the therapeutic potential that modulation of lipid metabolism can exert upon important aspects of glioma cell biology.

## 1. Introduction

Gliomas are primary tumors of the central nervous system (CNS) and are considered to be of glial origin. Diffuse gliomas are currently classified with emphasis on isocitrate dehydrogenase (IDH) mutation status. Astrocytomas with classic high-grade morphology and IDH wildtype are classified as glioblastomas (GBMs). Additionally, astrocytomas without classic GBM morphology, but with one or more of the genetic parameters of EGFR amplification, TERT promoter mutation, or gain of chromosome 7 with loss of chromosome 10 (+7/−10), are also classified as GBMs [1,2]. GBMs are the most common primary CNS tumors in adults (49.1% of all cases), with a mean incidence of 3.23 cases per 100,000 in the USA [3]. The average age at diagnosis of the disease is 64 and 65 years in Europe and the USA, respectively [3,4]. For patients over 40 years of age, 1-year survival is 40.6% and this value drops to 5.6% and 3.4% at 5 and 10 years after diagnosis [3].

Poor prognosis and low survival are related to several factors that make the treatment of high-grade gliomas difficult. Firstly, the location of the tumor often prevents total surgical resection of the lesion. Secondly, the tumor cells that remain after surgery have a high migratory and invasive capacity, causing tumor re-incidence close to the original site of surgical resection [5]. Another problem is the fact that post-surgery inflammation and reparative activity can serve as a stimulus for the proliferation, migration, and invasion of remaining cells [6]. Radiation therapy typically associated with post-surgical treatment of GBM causes inflammation in the brain that can also serve as a stimulus for remaining tumor cells. Lastly, most diffuse gliomas have an inadequate response to chemotherapy including the most commonly used drug, temozolomide (TMZ). Other chemotherapy options include BCNU (carmustine), CCNU (lomustine), PCV (Procarbazine, Lomustine, Vincristine), and bevacizumab. GBM can develop multidrug resistance (MDR) through the expression of proteins from the ABC transporter family, among others. Due to their ability to transport chemotherapeutic agents out of the tumor cell, these proteins may contribute to reduced treatment efficacy [7,8,9].

Polyunsaturated fatty acids (PUFAs) are known to induce apoptosis in many different tumor cells [10,11,12] and can reduce the expression of Ku80 involved in DNA repair as part of the heterodimer Ku70/Ku80 [11,13,14,15]. In brain tumors, docosahexaenoic acid (22:6 *n*−3) (DHA) has been reported to induce apoptosis, with evident PARP cleavage and TUNEL-positive cells, and to stimulate gene expression of multiple miRNAs related to the apoptotic process [16,17]. Gamma-linolenic acid (18:3 *n*−6) (GLA) has been shown to induce apoptosis and reduce tumor growth in a C6 glioma model in vivo [18].

There is considerable evidence that eicosapentaenoic acid (20:5 *n*−3) EPA and DHA improve the efficacy of chemotherapies including doxorubicin, vincristine and fludarabine, doxorubicin and cisplatin, carboplatin and vinorelbine or gemcitabine, 5-fluorouracil (5-FU), mitomycin C, epirubicin, arabinosylcytosine, tamoxifen, irinotecan, bortezomib, and docetaxel. Tumors include breast, colorectal, B cell leukemia, multiple myeloma, and non-small cell lung cancer and the results also show reductions in metastatic progression as well as reduced recurrence rates in some cases [19]. DHA has been found to sensitize glioma cells to chemotherapies including etoposide and CCNU [20,21], while EPA has been reported to sensitize glioma cells to TMZ and to improve radiation efficacy in glioma cells [19,22].

Previous studies have shown that in U87MG human glioma cells sensitive or resistant to TMZ, the presence of the PUFAs EPA or DHA has diverse additive or synergistic effects on the cells’ proliferative response to chemotherapy during the 72 h of exposure to PUFAs [23]. Exposure to PUFAs altered the fatty acid composition of cells and also altered the mRNA expression of genes important for the resistant phenotype including O6-methylguanine-DNA methyltransferase (MGMT), ABCB1, and some ABCC’s. Furthermore, there were changes in ABC transporter activity in fluorimetry assays studying the transport of fluorescent probes, which suggest that the presence of PUFAs could potentially modulate the MDR phenomenon in human glioma cells. Earlier studies have shown that the PUFAs GLA, DHA, and arachidonic acid (20:4 *n*−6) (AA) can alter miRNA expression in conditions of acute TMZ exposure, although the drug resistance status of the cells was not considered in these earlier studies [17].

In the present study, we have established several TMZ-resistant human glioma cell lines to further investigate the effects of EPA and DHA in the absence or presence of TMZ at concentrations which reflect achievable intratumoral TMZ concentrations, which are reported to range from 15 to 35 µm [24,25,26]. The cell response to PUFAs was assessed using proliferation and clonogenic assays, adhesion and transwell migration assays, glucose utilization and lactate production, fluorescent labeling with Nile red, propidium iodide, dihydroethidium and acridine orange, and phosphokinase proteome array analysis.

## 2. Results

### 2.1. Influence of EPA or DHA on Cell Number

**Figure 1:**
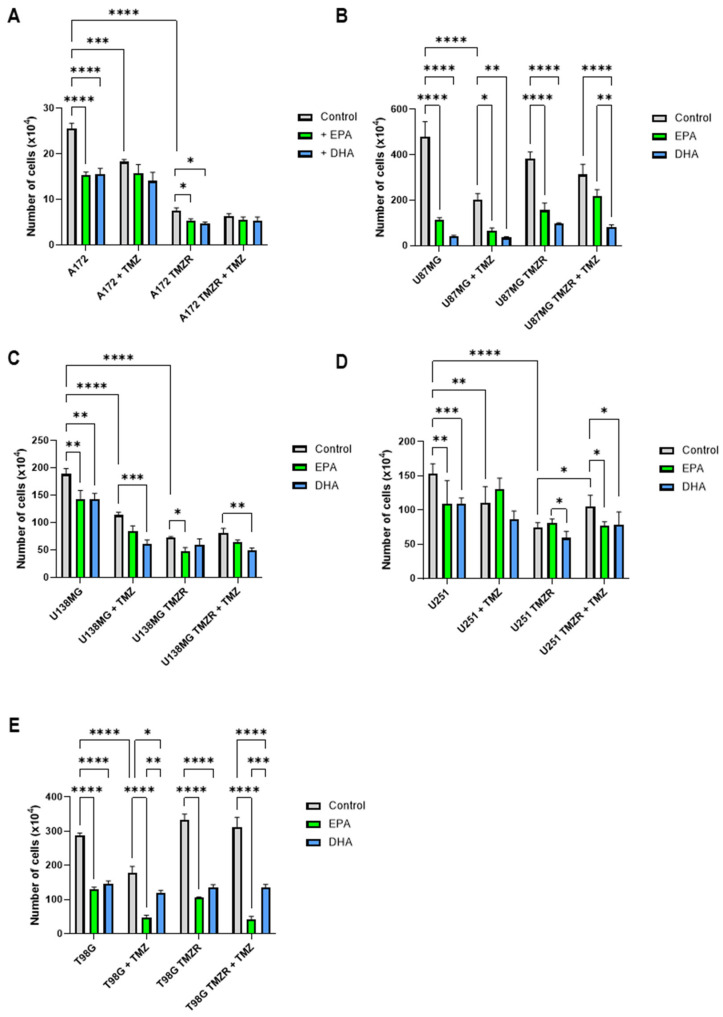
Effect of 100 µm EPA or DHA on cell number after 72 h in the presence or absence of 25 µm temozolomide (TMZ) for control or TMZ-resistant (TMZR) cells. (**A**) A172 and A172 TMZR. (**B**) U87MG and U87MG TMZR. (**C**) U138MG and U138MG TMZR. (**D**) U251 and U251 TMZ. (**E**) T98G and T98G TMZR. Data are presented as mean ± SEM, *N* = 4. Differences were considered significant at *p* < 0.05. * = *p* < 0.05; ** = *p* < 0.01; *** = *p* < 0.001; **** = *p* < 0.0001.

Initially, the effects of 25 µm TMZ were studied in TMZ-sensitive and TMZ-resistant (TMZR) human glioma cell lines (Figure 1). In addition, the effects of 72 h of exposure to 100 µm EPA or DHA on cell number were tested in the absence or presence of TMZ. A172 cell numbers were significantly reduced by both EPA (40.2%) and DHA (45.5%) in culture (Figure 1A). The presence of 25 µm TMZ caused a significant reduction in cell number (28.4%) and the additional presence of EPA or DHA had no significant effect. The TMZ-resistant cell line A172 TMZR was also affected by EPA and DHA, with a reduction in cell numbers of 30% and 36.7%, respectively (Figure 1A). The presence of 25 µm TMZ did not alter cell number significantly and nor did the additional presence of EPA or DHA.

U87MG cells were more sensitive to the effects of EPA or DHA than A172, with a reduction of 76.4% and 91.2%, respectively (Figure 1B). The presence of 25 µm TMZ caused a significant reduction in cell number (57.6%) and the additional presence of EPA or DHA caused a significant reduction of 68% and 82.8%, respectively. The TMZ-resistant cell line U87MG TMZR was also affected by EPA and DHA, with reduced cell numbers of 59.3% and 74.9%, respectively (Figure 1B). The presence of 25 µm TMZ did not alter cell number significantly and the additional presence of DHA caused a reduction of 74%, while EPA did not cause significant effects.

U138MG cells had reduced cell numbers in the presence of EPA (23.9%) or DHA (23.9%) (Figure 1C). The presence of 25 µm TMZ caused a significant reduction in cell number (39.9%) and the additional presence of DHA caused a significant reduction of 46.9%. The TMZ-resistant cell line U138MG TMZR was also affected by EPA, with a 33.3% reduction in cell number (Figure 1C). The presence of 25 µm TMZ did not alter cell number significantly and the additional presence of DHA caused a reduction of 41.8%.

U251 cell numbers were significantly reduced by both EPA (29.4%) and DHA (29.4%) (Figure 1D). The presence of 25 µm TMZ caused a significant reduction in cell number (28.1%) and the additional presence of EPA or DHA had no significant effect. The TMZ-resistant cell line, U251 TMZR, was unaffected by EPA or DHA (Figure 1D) and the presence of 25 µm TMZ did not reduce cell number. However, the additional presence of EPA or DHA caused a significant reduction in cell numbers of 26.9% and 25%, respectively.

Finally, the T98G cell line had reduced cell numbers in the presence of EPA (54.7%) or DHA (48.8%) (Figure 1E). The presence of 25 µm TMZ caused a significant reduction in cell number (37.6%) and the additional presence of EPA or DHA caused a significant reduction of 73.2% and 34.1%, respectively. The TMZ-resistant cell line T98G TMZR was also affected by EPA and DHA, with reduced cell numbers of 68.4% and 59.3%, respectively (Figure 1E). The presence of 25 µm TMZ did not alter cell number significantly but the additional presence of EPA or DHA caused a significant reduction in cell numbers of 86.5% and 57.1%, respectively.

### 2.2. Influence of TMZ, EPA, or DHA on Clonogenic Capacity

**Figure 2:**
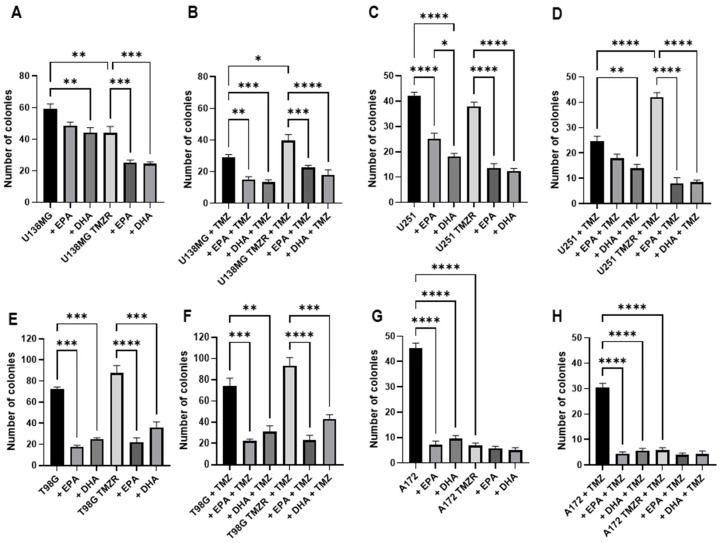
Effect of 100 µm EPA or DHA in the absence or presence of 25 µm temozolomide on the clonogenic capacity of control or TMZ-resistant cells. Colonies were counted using Image J software (version 1.54p). Data are presented as mean ± SEM, *N* = 3–4. Differences were considered significant at *p* < 0.05. * = *p* < 0.05; ** = *p* < 0.01; *** = *p* < 0.001; **** = *p* < 0.0001.

The effects of EPA or DHA on clonogenic capacity were studied and are presented in Figure 2. In U138MG cells DHA caused a significant reduction in clonogenic capacity, while both EPA and DHA caused a reduction in the U138MG TMZR cells (Figure 2A). In the presence of TMZ, EPA and DHA reduced capacity in both U138MG and U138MG TMZR cells (Figure 2B).

U251 cells had significantly reduced capacity in the presence of EPA or DHA, which was also seen for the U251 TMZR cells (Figure 2C). In the presence of TMZ, both EPA and DHA continued to cause a reduction in clonogenic capacity in U251 and U251 TMZR cells (Figure 2D).

The clonogenic capacity of T98G cells was reduced in the presence of EPA or DHA as was the capacity of T98G TMZR (Figure 2E). These effects remained when TMZ was present for both T98G and T98G TMZR (Figure 2F).

A172 had significantly reduced clonogenic capacity in the presence of EPA or DHA (Figure 2G), which was also seen in the presence of TMZ (Figure 2H). EPA and DHA tended to reduce clonogenic capacity in A172 TMZR, which did not reach significance. However, it should be noted that the clonogenic capacity of both cells was low in the presence of TMZ.

Colony areas were also calculated to compare the growth of colonies after successful formation and showed that the presence of EPA or DHA caused significant reductions in colony area in addition to reduced capacity for colony formation in the majority of cells (Supplementary Figure S1).

### 2.3. Influence of EPA or DHA on Cellular Adhesion and Spreading

**Figure 3:**
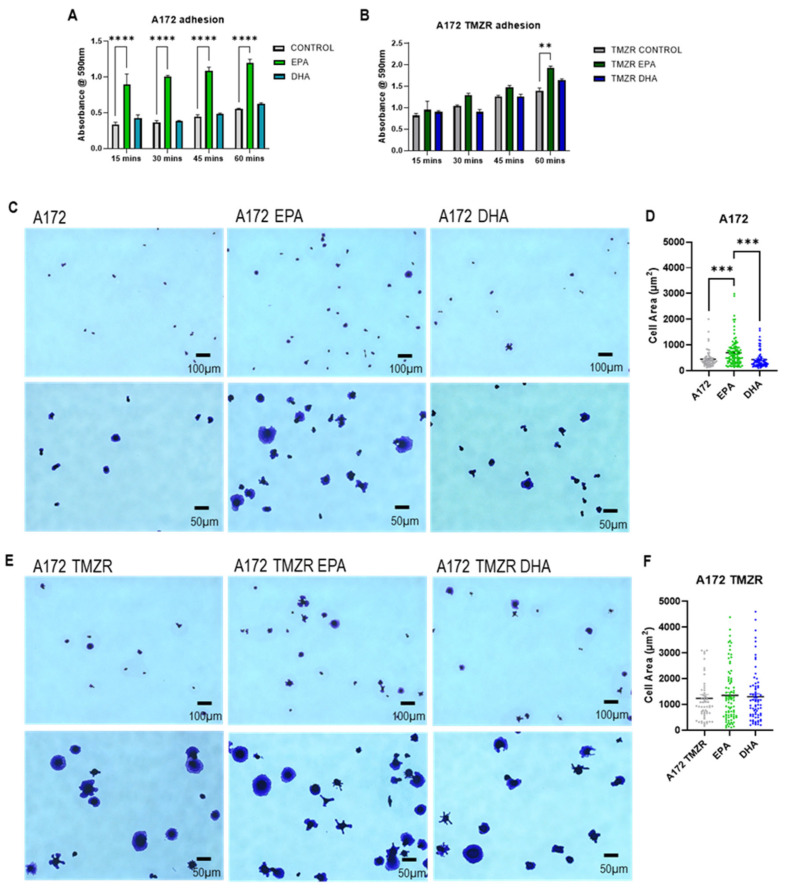
Effect of 100 µm EPA or DHA in the absence or presence of 25 µm temozolomide on adhesion and spreading of A172 and A172 TMZR cells. Images and cell area calculations are after 60 min adhesion. Data are presented as mean ± SEM, *N* = 3. Differences were considered significant at *p* < 0.05. ** = *p* < 0.01; *** = *p* < 0.001; **** = *p* < 0.0001.

To further understand the effects of PUFA exposure on glioma cell biology, cells were exposed to EPA or DHA for 72 h then tested for adhesion capacity. Pretreatment with EPA caused an increase in adhesion in A172 cells, which was not seen for DHA-treated cells (Figure 3A). No difference in adhesion was seen with EPA or DHA in A172 TMZR cells until the final time point, where EPA caused an increase in adhesion (Figure 3B). The spreading capacity of the cells was determined and morphological analysis clearly showed greater spreading by the A172 cells after pretreatment with EPA, which was not seen for DHA-pretreated cells (Figure 3C,D). No significant difference in cell spreading was seen for the A172 TMZR cells with EPA or DHA treatment, although the A172 TMZR cells had a significantly greater cell area than the A172 cells (Figure 3E,F).

**Figure 4:**
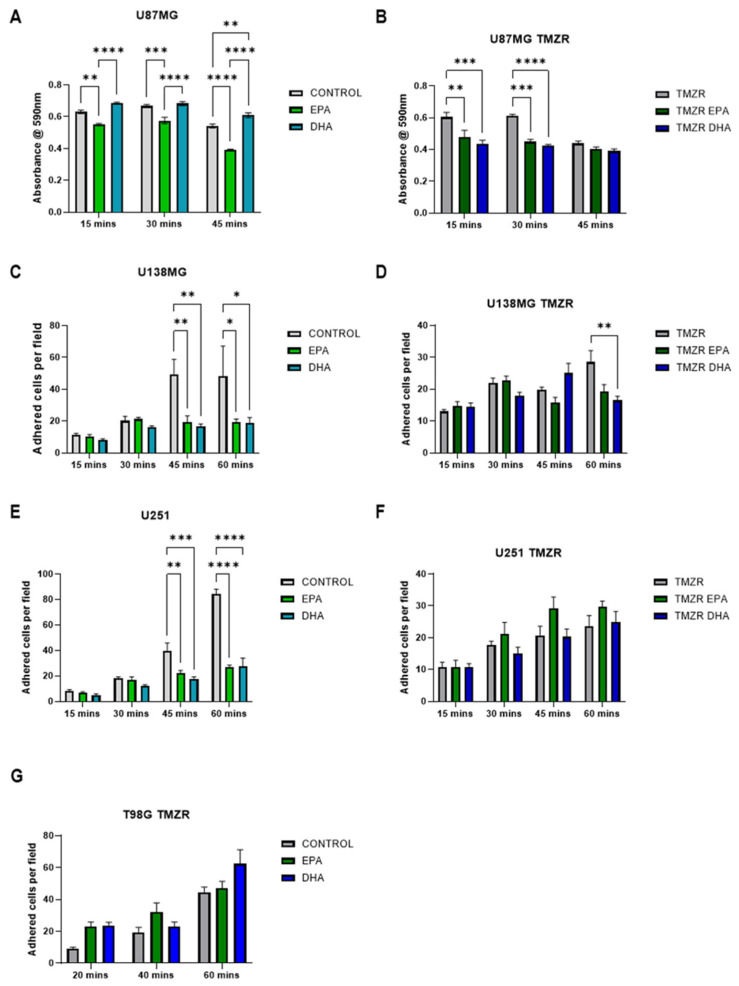
Effect of 100 µm EPA or DHA in the absence or presence of 25 µm temozolomide on adhesion of control or TMZ-resistant cells. (**A**) U87MG. (**B**) U87MG TMZR. (**C**) U138MG. (**D**) U138MG TMZR. (**E**) U251. (**F**) U251 TMZR. (**G**) T98G TMZR cells. Data are presented as mean ± SEM, *N* = 3–4. Differences were considered significant at *p* < 0.05. * = *p* < 0.05; ** = *p* < 0.01; *** = *p* < 0.001; **** = *p* < 0.0001.

In U87MG cells, EPA caused a reduction in adhesion while DHA had no significant effects (Figure 4A). Both EPA and DHA caused a significant reduction in adhesion in the U87MG TMZR cells (Figure 4B).

Both EPA and DHA caused significant reductions in adhesion in U138MG cells (Figure 4C), while only DHA caused a significant reduction in U87MG TMZR cells (Figure 4D).

U251 cell adhesion was reduced by both EPA and DHA pretreatment (Figure 4E). However, these effects were not seen in the U251 TMZR cells (Figure 4F).

Since the results for T98G and T98G TMZR cells are very similar for cell number and clonogenic capacity (Figure 1 and Figure 2), only the T98G TMZR cells were used in the adhesion assay. No significant difference in adhesion was seen after EPA or DHA pretreatment (Figure 4G).

### 2.4. Influence of EPA or DHA on Glioma Cell Migration

**Figure 5:**
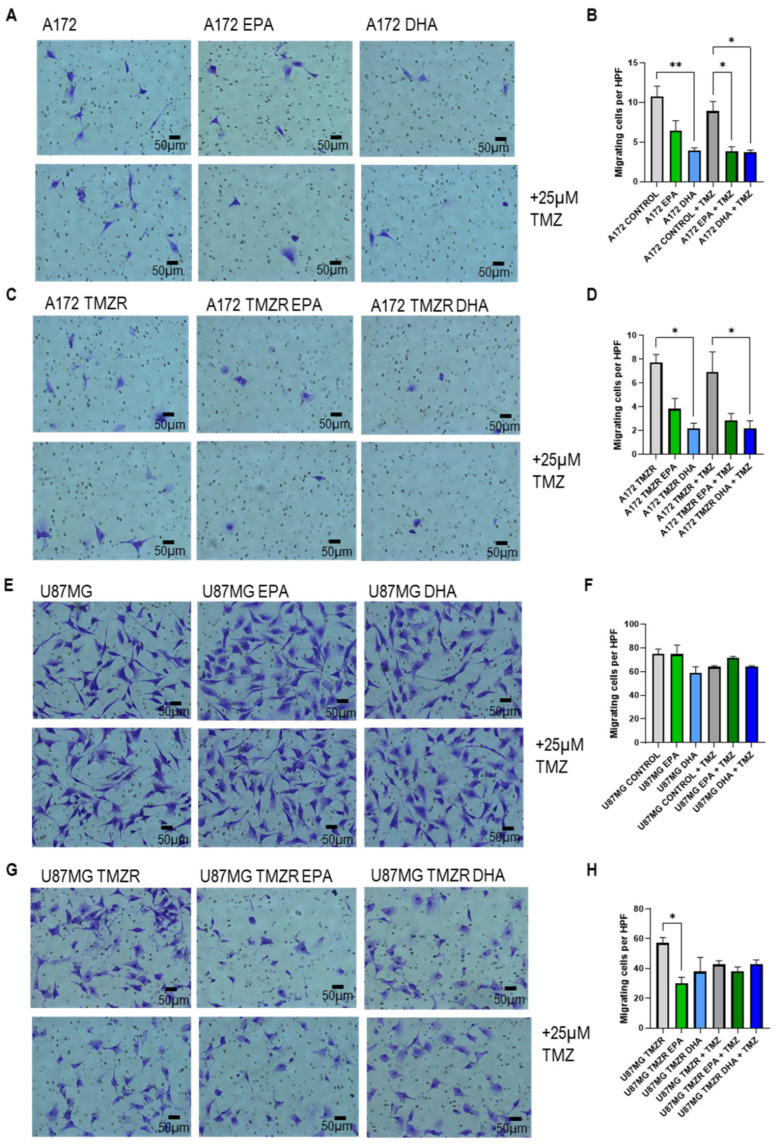
Effect of 100 µm EPA or DHA in the absence or presence of 25 µm temozolomide on migration of control or TMZ-resistant cells. (**A**,**C**,**E**,**G**) Images of migration assay. (**B**,**D**,**F**,**H**) Quantification of migrating cells per high power field image. Data are presented as mean ± SEM, *N* = 3. Differences were considered significant at *p* < 0.05. * = *p* < 0.05; ** = *p* < 0.01.

The effect of EPA or DHA exposure on glioma cell transwell migration was tested and the results are presented in Figure 5. A172 cells had significant inhibition of migration in the presence of DHA (Figure 5A,B). In the presence of TMZ, both EPA and DHA caused significant inhibition of A172 cell migration (Figure 5A,B). EPA tended to reduced migration in A172 TMZR cells while DHA caused a significant inhibition (Figure 5C,D). When TMZ was present, EPA again tended to inhibit and DHA caused a significant inhibition of A172 TMZR cell migration (Figure 5C,D). U87MG cell migration was unaffected by the presence of EPA or DHA, both in the absence and presence of TMZ (Figure 5E,F). In the case of U87MG TMZR cells, only EPA in the absence of TMZ had an inhibitory effect (Figure 5G,H).

**Figure 6:**
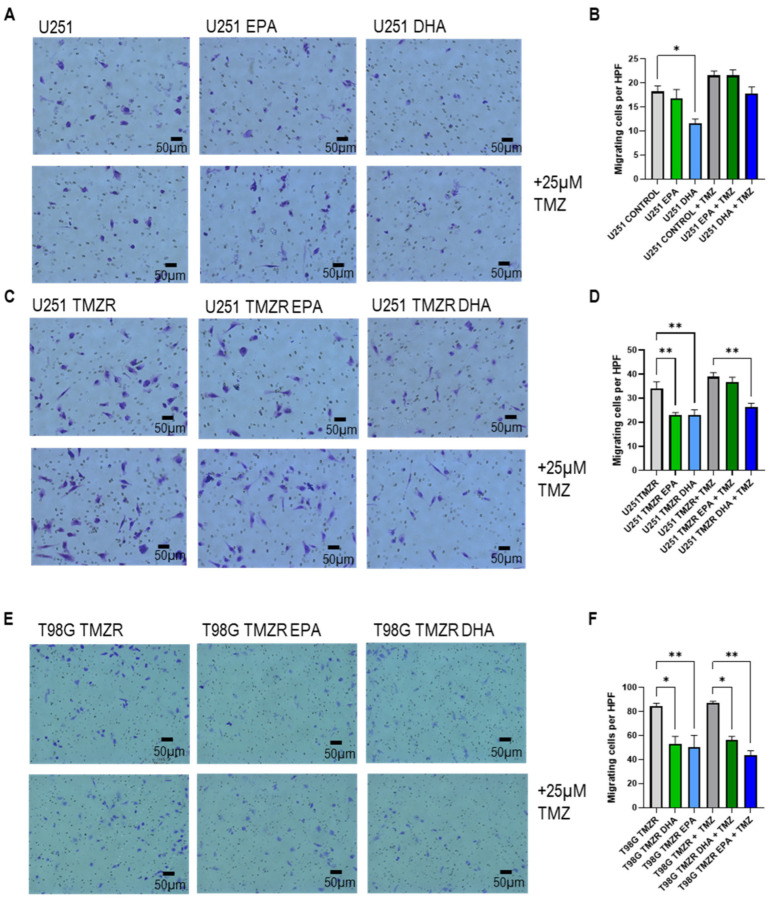
Effect of 100 µm EPA or DHA in the absence or presence of 25 µm temozolomide on migration of control or TMZ-resistant cells. (**A**,**C**,**E**) Images of migration assay. (**B**,**D**,**F**) Quantification of migrating cells per high power field image. Data are presented as mean ± SEM, *N* = 3–4. Differences were considered significant at *p* < 0.05. * = *p* < 0.05; ** = *p* < 0.01.

U251 cells displayed significant inhibition of migration in the presence of DHA (Figure 6A,B), which remained a tendency in the presence of TMZ but was no longer significant. Both EPA and DHA caused significant inhibition of U251 TMZR migration (Figure 6C,D). In the presence of TMZ, only DHA continued to cause inhibition of cell migration. T98G TMZR cell migration was significantly inhibited by EPA and DHA (Figure 6E,F). The inhibitory effect of both EPA and DHA was still seen in the presence of TMZ.

### 2.5. Alterations in Energy Metabolism Caused by EPA or DHA

**Figure 7:**
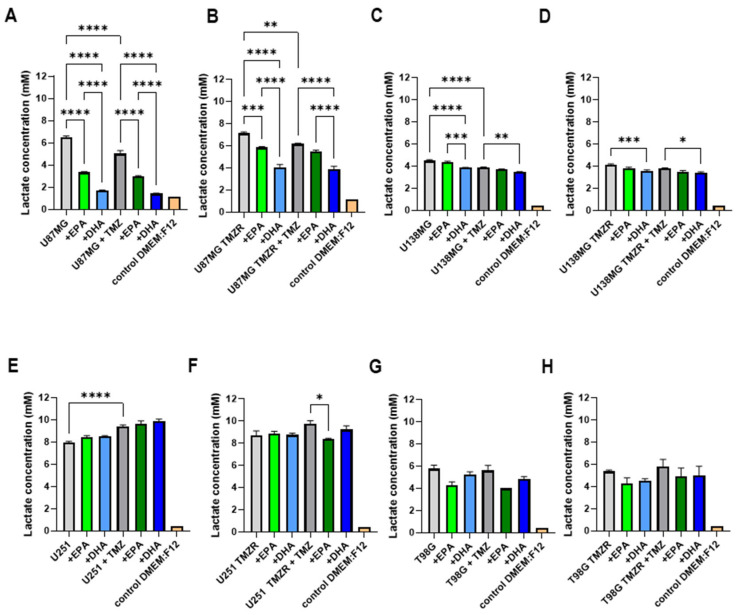
Effect of 100 µm EPA or DHA in the absence or presence of 25 µm temozolomide on lactate concentration in the medium of control or TMZ-resistant cells. (**A**) U87MG. (**B**) U87MG TMZR. (**C**) U138MG. (**D**) U138MG TMZR. (**E**) U251. (**F**) U251 TMZR. (**G**) T98G. (**H**) T98G TMZR cells. Data are presented as mean ± SEM, *N* = 4. Differences were considered significant at *p* < 0.05. * = *p* < 0.05; ** = *p* < 0.01; *** = *p* < 0.001; **** = *p* < 0.0001.

In order to test the effects of EPA or DHA on glioma cell metabolism, lactate and glucose concentrations were determined in the culture medium. Initially, the lactate concentration was analyzed and a decrease in lactate accumulation was evident in the medium of U87MG and U87MG TMZR in the absence or presence of 25 µm TMZ when EPA or DHA were present (Figure 7A,B). The U138MG strain also demonstrated effects of EPA and DHA on lactate accumulation in the medium (Figure 7C,D), but the U251 and T98G strains did not have significant results (Figure 7E,H).

**Figure 8:**
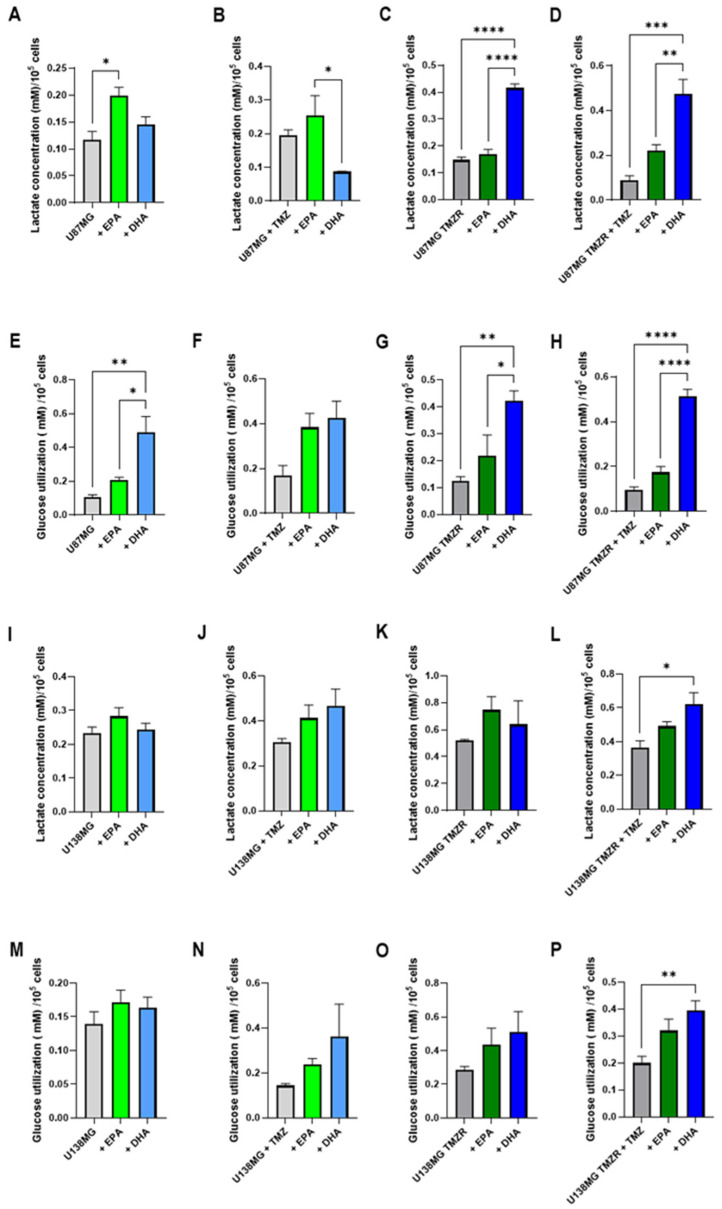
Effect of 100 µm EPA or DHA in the absence or presence of 25 µm temozolomide on lactate production and glucose consumption of control or TMZ-resistant cells. Lactate production for (**A**) U87MG; (**B**) U87MG + TMZ; (**C**) U87MG TMZR; (**D**) U87MG TMZR + TMZ; (**I**) U138MG; (**J**) U138MG + TMZ; (**K**) U138MG TMZR; (**L**) U138MG TMZR + TMZ. Glucose utilization for (**E**) U87MG; (**F**) U87MG + TMZ; (**G**) U87MG TMZR; (**H**) U87MG TMZR + TMZ; (**M**) U138MG; (**N**) U138MG + TMZ; (**O**) U138MG TMZR; (**P**) U138MG TMZR + TMZ. Data are presented as mean ± SEM, *N* = 4. Differences were considered significant at *p* < 0.05. * = *p* < 0.05; ** = *p* < 0.01; *** = *p* < 0.001; **** = *p* < 0.0001.

**Figure 9:**
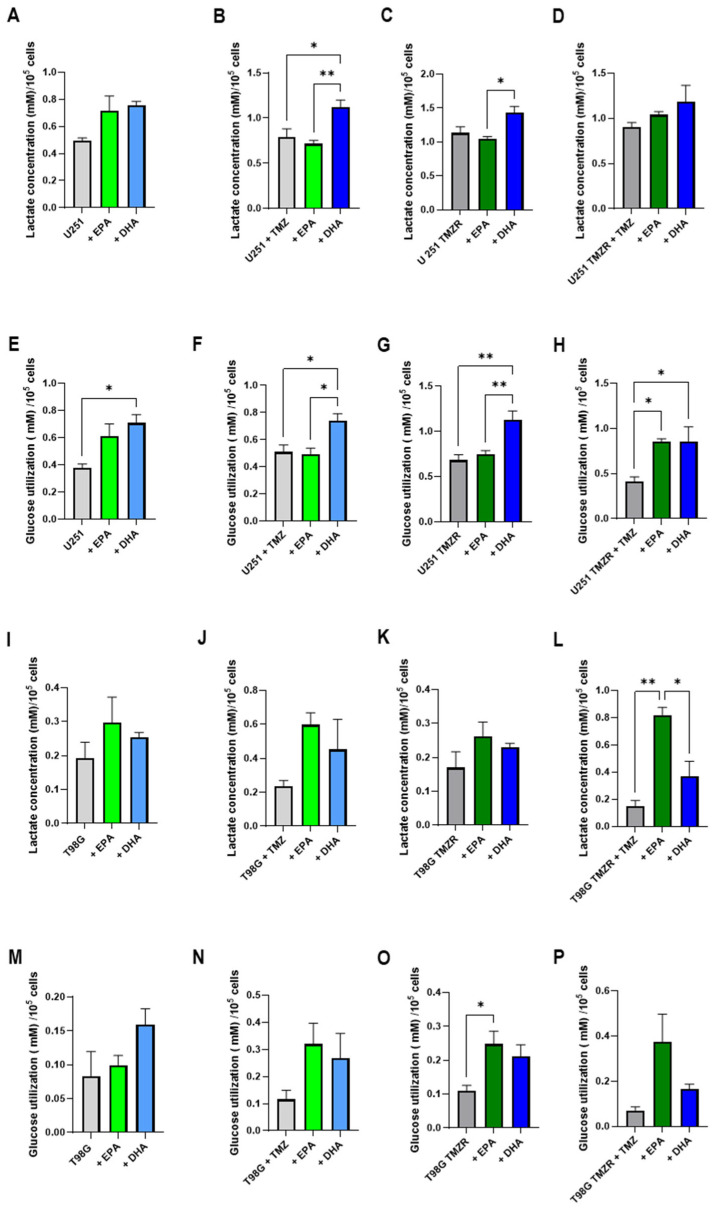
Effect of 100 µm EPA or DHA in the absence or presence of 25 µm temozolomide on lactate production and glucose consumption of control or TMZ-resistant cells. Lactate production for (**A**) U251; (**B**) U251 + TMZ; (**C**) U251 TMZR; (**D**) U251 TMZR + TMZ; (**I**) T98G; (**J**) T98G + TMZ; (**K**) T98G TMZR; (**L**) T98G + TMZ. Glucose utilization for (**E**) U251; (**F**) U251 + TMZ; (**G**) U251 TMZR; (**H**) U251 TMZR + TMZ; (**M**) T98G; (**N**) T98G + TMZ; (**O**) T98G TMZR; (**P**) T98G + TMZ. Data are presented as mean ± SEM, *N* = 4. Differences were considered significant at *p* < 0.05. * = *p* < 0.05; ** = *p* < 0.01.

When lactate production and glucose consumption were normalized by cell number, it was evident that EPA and DHA altered cell metabolism (Figure 8 and Figure 9). In some cases, EPA caused an increase in lactate production and an increase in glucose consumption (U87MG, T98G TMZR) (Figure 8A,E and Figure 9L,O), while DHA caused an increase in other cells lines: U87MG TMZR (Figure 8C,D,G,H), U138MG TMZR (Figure 8L,P), U251 (Figure 9B,F), U251 TMZR (Figure 9G,H). Similar results were seen for cells treated with EPA or GLA, including mouse (LLC-WRC256 and C6) and human (T24/83) tumor cells [10,27,28].

### 2.6. Lipid Accumulation, Cellular Damage, and Morphological Alterations Caused by EPA or DHA

**Figure 10:**
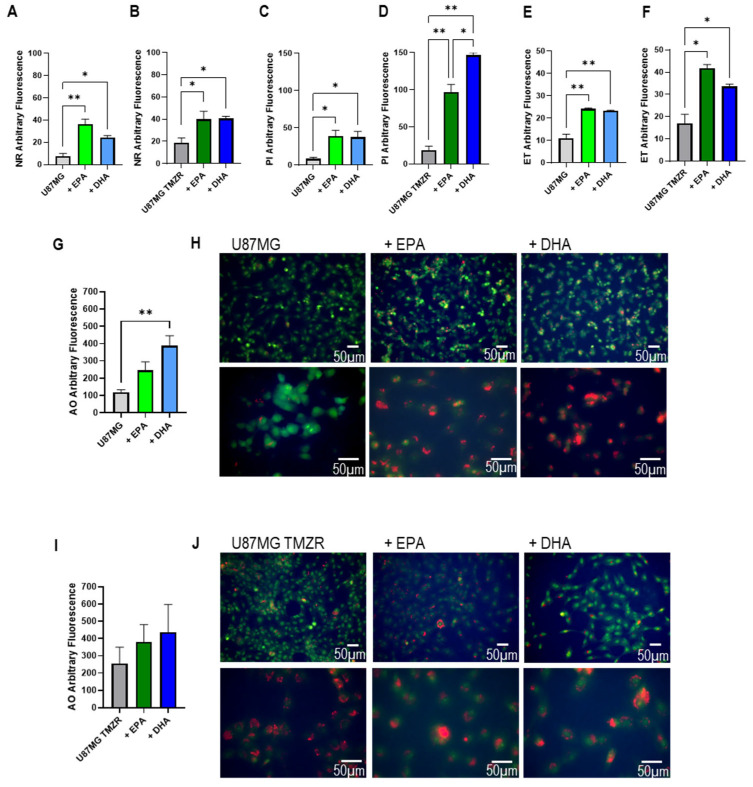
Effect of 100 µm EPA or DHA on Nile red, propidium iodide, dihydroethidium and acridine orange incorporation of U87MG or U87MG TMZR cells. (**A**) Nile red incorporation, U87MG; (**B**) Nile red incorporation, U87MG TMZR; (**C**) propidium iodide incorporation, U87MG; (**D**) propidium iodide incorporation, U87MG TMZR; (**E**) dihydroethidium incorporation, U87MG; (**F**) dihydroethidium incorporation, U87MG TMZR; (**G**) acridine orange fluorimetry (orange) U87MG; (**H**) acridine orange staining in U87MG cells; (**I**) acridine orange fluorimetry (orange) U87MG TMZR; (**J**) acridine orange staining in U87MG TMZR cells. (**H**,**J**) Upper row, low magnification; lower row, higher magnification. Data are presented as mean ± SEM, *N* = 6. Differences were considered significant at *p* < 0.05. * = *p* < 0.05; ** = *p* < 0.01.

Based on the results, U87MG and U87MG TMZR were chosen to further investigate the effects of EPA and DHA on cellular metabolism and morphology and the results are presented in Figure 10. The presence of EPA or DHA caused a significant increase in fluorescence from the neutral lipid probe Nile red in both U87MG and U87MG TMZR cells (Figure 10A,B). Plasma membrane permeability to propidium iodide was significantly increased in the presence of EPA or DHA in U87MG cells (Figure 10C) and more so in the U87MG TMZR cells (Figure 10D). The fluorescence of the reactive oxygen species probe dihydroethidium was increased in U87MG cells exposed to EPA or DHA (Figure 10E). The increase in fluorescence caused by EPA or DHA was greater in the U87MG TMZR cells (Figure 10F).

Acridine orange was used as a fluorescent probe for both nuclear labeling to observe apoptosis and to identify the acidic vesicle/lysosomal compartment of the cells. There was a tendency to increase lysosomal fluorescence in U87MG cells in the presence of EPA, but statistical significance was only observed in the presence of DHA (Figure 10G,H). In the case of U87MG TMZR, while a tendency to increase was observed, this did not reach statistical significance (Figure 10I,J).

**Figure 11:**
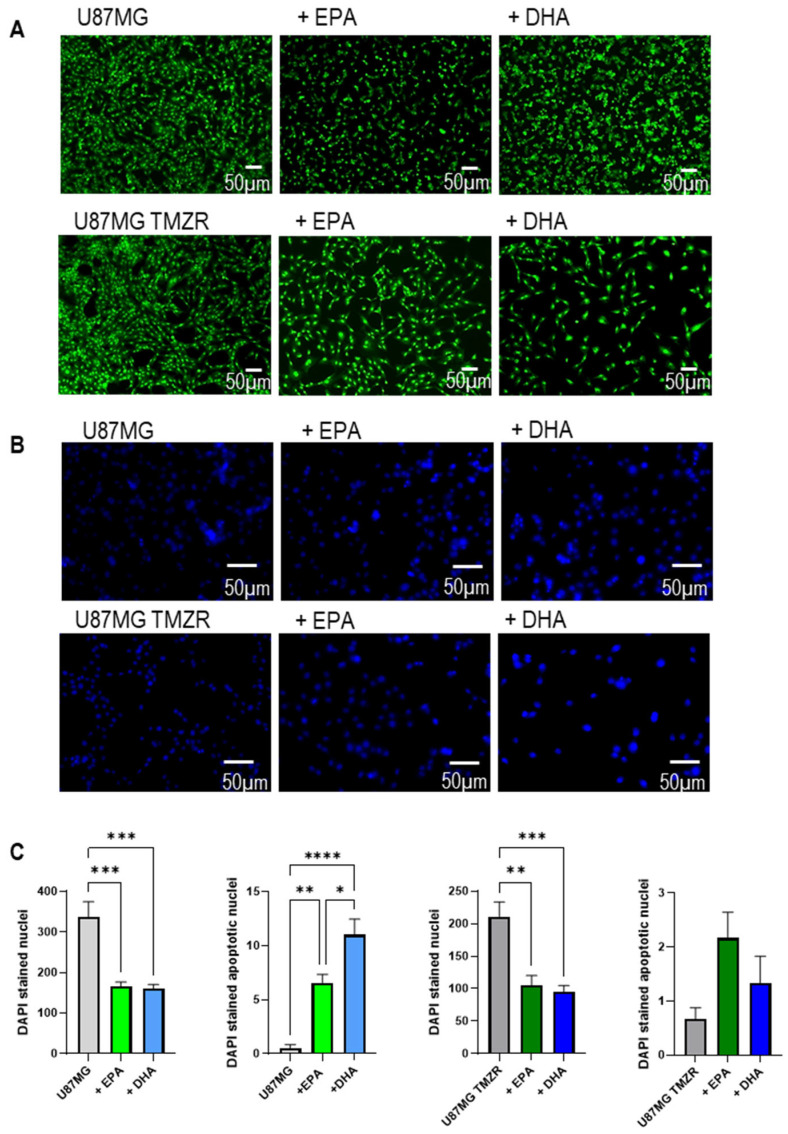
Effect of 100 µm EPA or DHA on acridine orange incorporation and DAPI staining of control or TMZ-resistant cells. (**A**) Acridine orange-stained cells; (**B**) DAPI-stained cells; (**C**) quantification of total DAPI-stained nuclei and DAPI-stained apoptotic nuclei for U87MG and U87MG TMZR. Data are presented as mean ± SEM, *N* = 6. Differences were considered significant at *p* < 0.05. * = *p* < 0.05; ** = *p* < 0.01; *** = *p* < 0.001; **** = *p* < 0.0001.

U87MG cells showed nuclear condensation typical of cells entering apoptosis in the presence of EPA or DHA and this was particularly evident in DHA-exposed cells stained with acridine orange (Figure 11A). U87MG TMZR cells had altered morphology, with larger cells than U87MG, and they showed less evidence of nuclear condensation (Figure 11A). Similar results were seen for DAPI labeling of nuclei, where reduced total stained nuclei and increased nuclear condensation typical of apoptosis was seen in the presence of EPA or DHA (Figure 11B,C).

### 2.7. Human Phosphokinase Proteome Array

**Figure 12:**
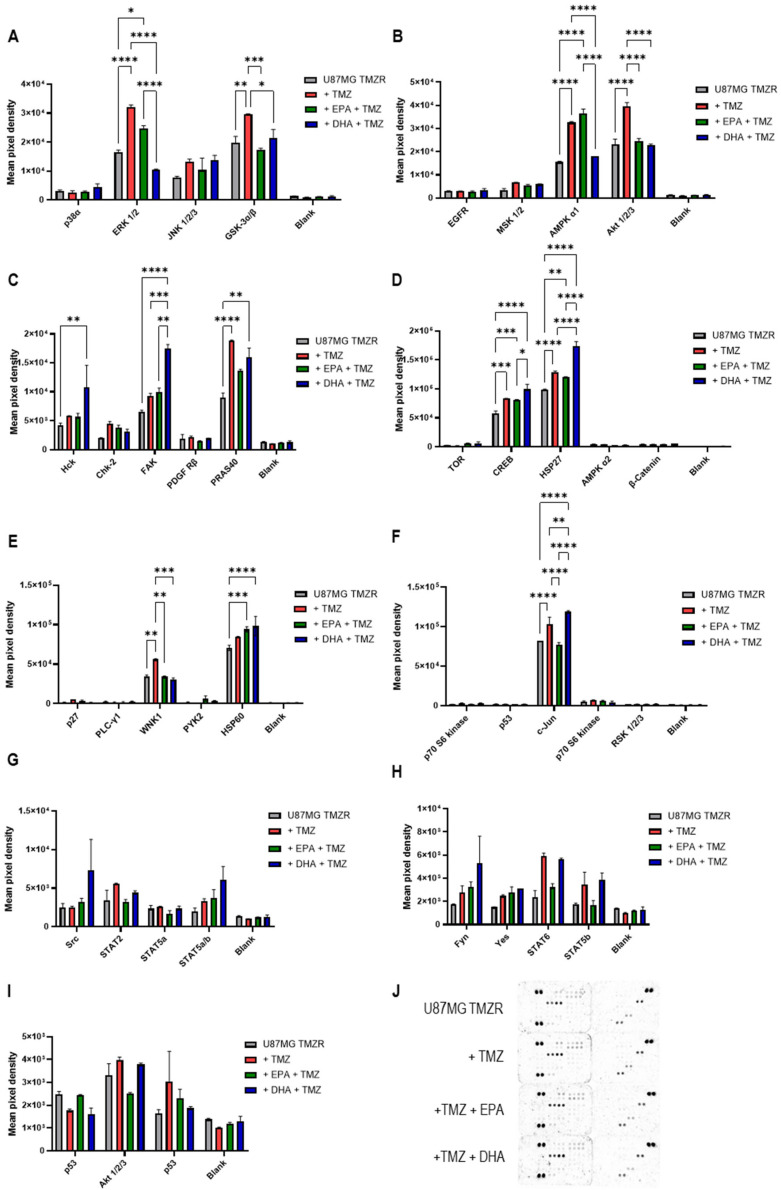
Effect of 100 µm EPA or DHA on phosphoprotein profiles of U87MG TMZR cells in the presence of 25 µm temozolomide. (**A**) p38α, ERK1/2, JNK1/2/3, GSK3α/β; (**B**) EGFR, MSK1/2, AMPKα1, Akt1/2/3; (**C**) Hck, Chk-2, FAK, PDGF Rβ, PRAS40; (**D**) TOR, CREB, HSP27, AMPK α2, β-catenin; (**E**) p27, PLC-γ1, WNK1, PYK2, HSP60; (**F**) p70 S6 kinase, p53, c-Jun, p70 S6 kinase, RSK1/2/3; (**G**) Src, STAT2, STAT5a, STAT5a/b; (**H**) Fyn, Yes, STAT6, STAT5b; (**I**) p53, Akt1/2/3, p53; (**J**) image of membranes, see Supplementary Figure S2 for further information. Data are presented as mean ± SEM, *N* = 2. Differences were considered significant at *p* < 0.05. * = *p* < 0.05; ** = *p* < 0.01; *** = *p* < 0.001; ****= *p* < 0.0001.

The effects of EPA or DHA on signaling pathways were studied in U87MG TMZR cells using a human phosphokinase proteome array and the results are presented in Figure 12. The presence of TMZ caused a significant increase in phospho-ERK 1/2 (T202/Y204, T185/Y187) and in phospho-GSK 3α/β (S21/S9) (Figure 12A). No effect of TMZ was seen for phospo-p38α (T180/Y182) or phospho-JNK 1/2/3 (T183/Y185, T221/Y223). When comparing TMZ alone with TMZ and EPA or TMZ and DHA, the presence of EPA caused a significant reduction in phospho-GSK 3α/β. The presence of DHA caused a significant reduction in phospho-ERK 1/2 and in phospho-GSK 3α/β.

The presence of TMZ caused a significant increase in phospho-AMPK α1(T183) and phospho-Akt 1/2/3 (S473) (Figure 12B). No effect of TMZ was seen for phospho-EGFR (Y1086) or phospho-MSK 1/2 (S376/S360). Comparing TMZ with TMZ and EPA or DHA, EPA caused a significant decrease in phospho-Akt 1/2/3, while DHA caused a significant decrease in phospho-AMPK α1 and phospho-Akt 1/2/3.

The presence of TMZ caused a significant increase in phospho-PRAS40 (T246) (Figure 12C). No effect of TMZ was seen for phospho-Hck (Y411), phospho-Chk-2 (T68), or phospho-PDGF Rβ (Y751). Comparing TMZ with TMZ and EPA or DHA, DHA caused a significant increase in phospho-FAK (Y397).

The presence of TMZ caused a significant increase in phospho-CREB (S133) and phospho-HSP27 (S78/S82) (Figure 12D). No effect of TMZ was seen for phospho-TOR (S2448), phospho-AMPK α2 (T172), or β-catenin. Comparing TMZ with TMZ and EPA or DHA, DHA caused a significant increase in phospho-HSP27.

The presence of TMZ caused an increase in phospho-WNK1 (Figure 12E) which was decreased in the presence of EPA or DHA. No effect of TMZ was seen for phospho-p27 (T198), phospho-PLC-γ1 (Y783), phospho-PYK2 (Y402) or HSP60. However, HSP60 was increased in the presence of TMZ and EPA or DHA when compared with U87MG TMZR cells alone.

The presence of TMZ caused a significant increase in phospho-c-Jun (S63) (Figure 12F). Comparing TMZ with TMZ and EPA or DHA, EPA caused a significant decrease in phospho-c-Jun while DHA caused a significant increase in phospho-c-Jun. No effect of TMZ was seen for phospho-p70 S6 kinase (T389 or T421/S424), phospho-p53 (S392), or RSK 1/2/3 (S380/S386/S377).

The phosphorylation profiles of Src (Y419), STAT 2 (Y689), STAT 5a (Y694), STAT 5a/b (Y694/Y699), Fyn (Y420), Yes (Y426), STAT 6 (Y641), STAT 5b (Y699), p53 (S46 and S15)s and Akt 1/2/3 (T308) were not significantly altered by TMZ or TMZ in the presence of EPA or DHA (Figure 12G–I).

## 3. Discussion

Each of the human glioma cells, when grown in the presence of 25 µm TMZ, had reduced cell numbers in comparison with the control, ranging from 28 to 57%. Each of the TMZ-resistant cells used in this study had a significantly increased IC50 for TMZ in comparison with the parent cell lines and, proving the acquisition of TMZ resistance; none of the TMZR cells had altered cell numbers in the presence of TMZ. The T98G and T98G TMZR cells had similar responses, showing the intrinsic TMZ resistance of this cell line, which is known to express unmethylated MGMT [29]. All of the TMZ-sensitive cells were significantly affected by the presence of 100 µm EPA or DHA, with reduced cell numbers ranging from 23 to 76% and 24–91%, respectively. In the presence of TMZ, EPA or DHA caused further reductions in cell numbers for U87MG, U138MG, and T98G cells. In the case of the TMZR cells, U87MG TMZR and T98G TMZR were the most affected by EPA or DHA, both in the absence or presence of TMZ. While most of the effects of EPA or DHA in the presence of TMZ were additive in the cell lines studied, DHA had a synergistic, enhancing effect on reduction in cell number in the presence of TMZ in U138MG TMZR and U251 TMZR cells. In T98G TMZR and U251 TMZR cells, EPA had a synergistic, enhancing effect on reduction in cell number in the presence of TMZ. It is important to note that the concentrations used in this study reflect achievable intratumoral TMZ concentrations which are reported to range from 15 to 35 µm [24,25,26], while fatty acids can reach concentrations from 100 to 700 µm in plasma and tissues after supplementation [30,31]. Both EPA and DHA are readily taken up by the brain and PUFA supplementation is considered safe in doses up to 3–5 g/day [32,33]. Animal models of glioma have also shown that PUFAs such as GLA and DHA can readily enter the brain and alter the fatty acid composition of tumor tissue [18,34].

In order to test the difference in survival ability between parent cells and their TMZR derived cells, a clonogenic assay was performed in the presence of EPA or DHA. In all of the TMZ-sensitive cells the presence of EPA or DHA significantly reduced the clonogenic capacity and the TMZR cells were also inhibited by the presence of EPA or DHA. When the assay was performed in the presence of TMZ, both the TMZ-sensitive and TMZR cells were again inhibited in the presence of EPA or DHA. In the case of the U138MG TMZR and U251 TMZR cells, DHA had a synergistic, enhancing effect on the inhibition of colony formation in the presence of TMZ. Similarly, in U251 TMZR, T98G TMZR, and A172 TMZR cells, EPA had a synergistic, enhancing effect on the inhibition of colony formation in the presence of TMZ.

It is important to point out that many previous studies have shown that PUFAs such as EPA and DHA, at concentrations similar to those used in the present study, do not have adverse effects on normal brain cells such as neurons and astrocytes [35,36,37]. Indeed, DHA is known to contribute to normal astrocyte maturation, differentiation, and function, while EPA and DHA have protective effects in the astrocytic response to cytokine challenge [38,39,40].

These initial results showing inhibitory effects of EPA or DHA raised the question of whether the pretreatment of cells with EPA or DHA could interfere with cell adhesion, which is an important factor in cell survival. The effects of EPA or DHA were variable among cell lines, with A172 cells having increased adhesion in the presence of EPA, while, in contrast, EPA caused a significant reduction in adhesion in the U87MG, U138MG, and U251 cells. DHA caused a significant reduction in adhesion in the U138MG and U251 cells. These effects were considerably reduced in the TMZR cells, with EPA increasing adhesion in the A172 TMZR cells, EPA and DHA causing inhibition in the U87MG TMZR cells, and DHA causing inhibition in the U138MG TMZR cells. The variable effects of EPA or DHA on cell adhesion may be related to the heterogeneous expression of cell adhesion molecules, such as integrins, among the cell lines, which has previously been linked to radio sensitivity in several of these cell lines [41,42,43].

The next step in the study was to identify possible effects of EPA or DHA on glioma cell migratory capacity. Using the transwell migration assay, we identified variable effects in the different cell lines with A172 cell migration inhibited by DHA, with a tendency of inhibition by EPA. In the presence of TMZ, both EPA and DHA caused significant inhibition of migration in the A172 cells. A172 TMZR cell migration was inhibited by DHA in the absence and presence of TMZ, while EPA showed a tendency to inhibit migration which did not reach statistical significance.

The U87MG cells were unaffected by EPA or DHA under any condition, while the U87MG TMZR cells’ migration was only inhibited by EPA in the absence of TMZ. The U251 cells had significantly reduced migration with DHA in the absence of TMZ, while the U251 TMZR cells were inhibited by EPA in the absence of TMZ and inhibited by DHA in both the absence and presence of TMZ. T98G TMZR cell migration was significantly inhibited by both EPA and DHA in the absence and presence of TMZ. Overall, in the present study, DHA had a more inhibitory effect on migration then EPA.

EPA has previously been reported to inhibit cell migration in various tumors including esophageal and breast tumors [44,45]. Both EPA and DHA inhibit breast tumor cell invasion through matrigel, with DHA having a greater effect than EPA [45]. DHA has been shown to suppress expression of EMT-related genes including SNAIL, SLUG, ZEB1, and ZEB2 in colorectal cancer cells, resulting in decreased expression of N-cadherin and vimentin, increased E-cadherin expression, and reduced cell migration [46]. Similar findings have been seen in breast cancer, with DHA decreasing metalloproteases such as MMP2 and MMP9 as well as vimentin and reducing cell migration [47,48]. DHA has been reported to interfere with membrane composition and migratory capacity in GBM cells which express fatty acid binding protein 7 (FABP7). It was proposed that when FABP7 is bound to DHA, it leads to the induction of membrane remodeling associated with the inhibition of GBM cell migration [49].

To test the effects of EPA or DHA on metabolism, we measured lactate accumulation and glucose utilization in the culture medium. EPA and DHA caused significant reductions in lactate accumulation in the medium of U87MG cells in the absence and presence of TMZ. Similar effects were seen on lactate accumulation in the U87MG TMZR cells. When lactate production and glucose consumption were normalized by cell number, it was evident that EPA and DHA altered cell metabolism, with increases in glucose consumption and lactate production seen for various cell lines. The variable response to EPA or DHA among the different cell lines may be related to metabolic preferences. Previous studies have shown that the cell lines used in the present study have differing metabolic profiles and differing responses to substrate availability particularly in relation to glycolysis and fatty acid oxidation [50,51,52,53]. While TMZ has been reported to cause an increase in saturated fatty acid (palmitic acid) uptake and oxidation in GBM stem cells [54], it appears that increased EPA or DHA uptake does not cause similar effects and results in increased glucose utilization and lactate production. Similar findings have been reported in several cell lines including mouse (LLC-WRC256 and C6) and human (T24/83) tumor cells [11,27,28]. EPA has also been reported to increase lactate production in normal muscle cells [55].

Using U87MG and U87MG TMZR cells as a representative model we found significant increases in Nile red fluorescence in the presence of EPA or DHA, indicative of an increase in lipid droplet content in the cells, as previously reported in [23]. Plasma membrane permeability was also increased by EPA or DHA treatment, seen by the increase in propidium iodide fluorescence in both U87MG and U87MG TMZR cells. In addition to increased lipid droplet accumulation and increased membrane permeability, EPA and DHA both caused significant increases in dihydroethidium conversion to ethidium and subsequent increases in fluorescence signal in U87MG and U87MG TMZR cells. This indicates an increase in oxidative stress in the PUFA-exposed cells, which has also been described in other tumor cells [11,27,28,56]. Several studies using natural compounds such as curcumin, resveratrol, and steroidal saponins together with TMZ have also reported additive or synergistic effects including ROS generation and increased oxidative stress [57]. Acridine orange labeling of the cells showed alterations in morphology, with marked nuclear condensation typical of cells entering apoptosis being particularly evident in U87MG cells exposed to EPA or DHA. The acidic vesicular organelle compartment appeared to be significantly increased in the U87MG cells in the presence of DHA with a tendency to increase in the presence of EPA; however, this was not seen for the U87MG TMZR cells. These findings are corroborated by previous studies in U87MG, U251, and D54MG glioma cells, where the presence of DHA at increasing concentrations from 20 to 50 µm caused an increase in the autophagy marker LC3B-II [16]. The U87MG TMZR cells appeared to contain a higher number of acidic vesicles than the parental line, and previous studies have reported this in U251 glioma cells [58]. It will be important to identify whether these changes reflect alterations in autophagic processes in future studies.

**Figure 13:**
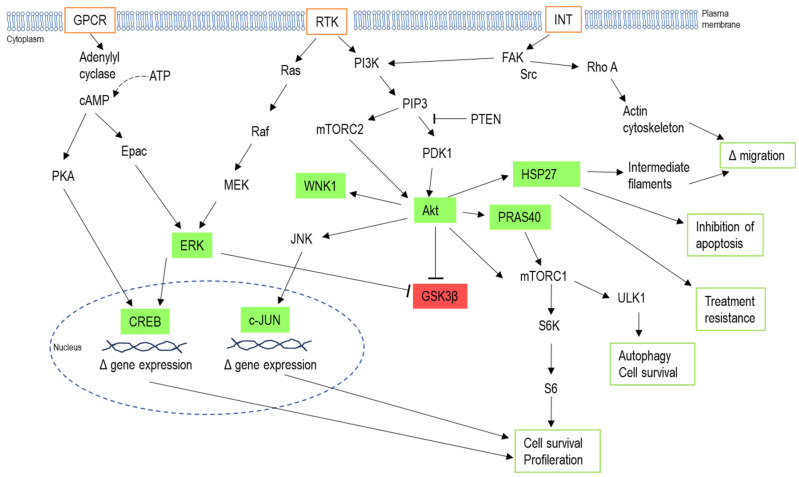
Outline of phosphoproteins significantly altered by the presence of temozolomide (TMZ) in U87MG TMZR cells. The proteins in green are activated when phosphorylated—CREB, ERK, Akt, WNK, HSP27, PRAS40. The protein in red, GSK3β, is inactivated when phosphorylated. Pathways influenced by these proteins are related to cell survival, resistance to apoptosis, control of cell migration, and chemotherapy treatment resistance. Akt—Akt kinase or protein kinase B; ATP—adenosine triphosphate; cAMP—cyclic adenosine monophosphate; c-JUN—cellular homolog of viral JUN; CREB—cAMP response element binding protein; Epac—exchange protein activated by cAMP; ERK—extracellular signal regulated protein; FAK—focal adhesion kinase; GPCR—G protein coupled receptor; GSK3β—glycogen synthase kinase 3 beta; HSP27—heat shock protein 27; INT—integrin; JNK—JUN terminal kinase; MEK—mitogen-activated protein kinase kinase; mTORC1 or 2—mammalian target of rapamycin complex 1 or 2; PDK1—phosphoinositide-dependent kinase 1; PI3K—phosphoinostide-3-kinase; PIP_3_—phosphatidylinositol-3,4,5-triphosphate; PKA—protein kinase A; PRAS40—proline-rich Akt substrate of 40KDa; PTEN—phosphatase and tensin homolog; Raf—serine/threonine protein kinase Raf; Ras—serine/threonine protein kinase Ras; Rho A—Rho GTPase A; RTK—receptor tyrosine kinase; S6—ribosomal protein S6; S6K—ribosomal protein S6 kinase; Src—non-receptor tyrosine kinase Src; ULK1—UNC-51-like autophagy-activating kinase 1; WNK1—with no lysine kinase 1.

The data obtained from the phosphokinase proteome assay pointed to several important changes in the protein phosphorylation state of U87MG TMZR cells in response to the presence of TMZ. Increases in phosphorylated ERK 1/2, GSK 3α/β, AMPK α1, Akt 1/2/3, PRAS40, CREB, HSP27, WNK1, and c-Jun were identified in the presence of TMZ. The cellular response in favor of survival and inhibition of cell death was evident, leading to effective resistance to TMZ. These changes and their role in intracellular signaling are summarized in Figure 13.

Previous studies have shown that TMZ causes an increase in ERK phosphorylation in U87MG cells and C6 glioma cells [59,60]. In other studies, U87MG TMZR cells were reported to have increased Akt, GSK3β, and PRAS40 phosphorylation in comparison with their parental TMZ sensitive line [61]. In a different approach, CREB was shown to be important for TMZR in several glioma cell lines by using miR-433-3p to increase chemosensitivity of glioma cells to TMZ by targeting CREB expression [62]. In a study of HSP response to TMZ, glioma cells were found to increase HSP 27, 70, and 90 expression, while HSP 60 remained unchanged. However, the phosphorylation state of the proteins was not determined [63]. In primary GBM cells and in mouse glioma, TMZ caused an increase in WNK1 phosphorylation [64,65]. Exposure to TMZ was also shown to increase c-Jun phosphorylation and participate in the development of TMZ resistance [66].

When U87MG TMZR cells in the presence of TMZ were exposed to EPA, the phosphorylation of GSK 3α/β, Akt 1/2/3, WNK1, and c-Jun was decreased. When U87MG TMZR cells in the presence of TMZ were exposed to DHA, the phosphorylation of ERK 1/2, GSK 3α/β, AMPK α1, Akt 1/2/3, and WNK1 was decreased. However, when U87MG TMZR cells in the presence of TMZ were exposed to DHA, the phosphorylation of FAK, HSP27, and c-Jun was increased. When U87MG TMZR cells were exposed to TMZ and EPA or TMZ and DHA, HSP60 expression was increased in comparison with U87MG TMZR alone. Previous studies have reported that 50 µm DHA caused a significant reduction in p-Akt (S473) in U87MG cells [16].

These data show that both EPA and DHA inhibit the phosphorylation of several proteins which have increased phosphorylation in U87MG TMZR cells in response to TMZ. Together with the phosphorylation profiles in various glioma cells mentioned previously, our data support the hypothesis that these proteins play an important role in the development of TMZR in these cells and that the presence of EPA or DHA can reduce TMZR by altering their phosphorylation profiles.

Despite studies showing that EPA and DHA improve the efficacy of chemotherapies in several tumor types, there is a paucity of data showing similar effects in relation to TMZ. There are limitations to the study which should be addressed in the future, including by longer periods of exposure to TMZ, which may allow the identification of higher rates of apoptosis and of senescence occurring in the cells. Also, since cell counts were determined, bromodeoxyuridine incorporation assays would permit further analysis of the proliferative rate of the cells under the different experimental conditions. Finally, the use of patient-derived primary cells in 3D culture will also be an important approach in future studies.

Despite its limitations, the present study highlights the alterations which occur in TMZR cells when exposed to EPA or DHA and builds on the findings previously reported in the U87MG TMZR model, where these PUFAs were shown to interfere with drug resistance, in part through modulation of ABC transporter proteins [23]. Thus, the present study, together with our previous work, provides evidence that EPA and DHA can significantly enhance TMZ efficacy in TMZR GBM cell lines with diverse genetic backgrounds. These findings, together with the known safety record of EPA and/or DHA supplementation in humans, provide initial support for the proposal of in vivo testing in combination with TMZ in patients.

Future studies will investigate the downstream signaling mechanisms involved in PUFA modulation of TMZ resistance in established glioma cells and in patient-derived primary cells in both 2D and 3D culture conditions. In vivo glioma models where TMZ and PUFAs can be administered concomitantly either orally or by osmotic pump infusion will also be used to test combination therapies, as previously reported in GLA-treated glioma models [18,67], in the search for further actionable target proteins related to TMZR.

## 4. Materials and Methods

### 4.1. Cell Culture

A172, U87MG, U138MG, U251, and T98G human glioblastoma cells were cultured in DMEM:F12 (Dulbecco’s Modified Eagle Medium and F12—Gibco Inc., Thermo Fisher Scientific, Grand Island, NY, USA) 1:1, supplemented with 10% (*v*/*v*) FBS (fetal bovine serum—Life Technologies, São Paulo, SP, Brazil), 50 units/mL penicillin, and 50 μg/mL streptomycin. The cells were originally obtained from the ATCC and were regularly checked for mycoplasma by Hoechst 33342 staining and PCR analysis. Cells were maintained at 37 °C in a humidified atmosphere with 5% CO_2_ in 25 or 75 cm^2^ flasks, until the desired confluency. Cells were washed with phosphate-buffered saline (PBS), pH 7.2, and trypsinized (trypsin 0.025%/EDTA 0.02%) for further use in experiments.

### 4.2. Establishing the Temozolomide Drug-Resistant Cell Lines

Cells were grown in culture medium, as detailed in Section 4.1, in 24-well plates at a cell density of 3 × 10^4^ cells/well. Cells were exposed to gradually increasing concentrations of temozolomide (TMZ) (Cayman Chemical, Ann Arbor, MI, USA), starting at 1 μm and reaching the previously published IC50 of 25 μm [68], with fresh medium and TMZ added every 48 h and weekly trypsinization of cells to expand the cultures. Similar IC50 values have been reported for these cells and other GBM cell lines [69,70].

The treatment scheme was as follows: 0–7 days, 1 μm; 8–14 days, 2 μm; 15–21 days, 4 μm; 22–28 days, 8 μm; 29–35 days, 10 μm; 36–45 days, 15 μm; 46–60 days, 20 μm; 61–90 days, 25 μm [10]. After 90 days, cells were well adapted to 25 μm TMZ and stocks were frozen in DMEM/10%FBS/10% DMSO (Sigma Aldrich, São Paulo, SP, Brazil) and stored in liquid nitrogen for use in all future experiments. The IC_50_ values for TMZ were determined following the method previously described [23] and calculated using Graph Pad Prism 10 (GraphPad Prism 10 Software, San Diego, CA, USA). The IC_50_ values obtained were as follows: A172 = 36.1 µm, A172 TMZR = 137.5 µm; U87MG = 23.6 µm, U87MG TMZR = 94.3 µm; U138MG = 6.4 µm U138MG TMZR = 27.6 µm; U251 = 9.8 µm U251 TMZR = 27.2 µm; T98G > 500 µm T98G TMZR > 500 µm.

### 4.3. Cell Counts Assay

Control and TMZR cells were seeded at 3 × 10^4^ cells/well in 24-well plates and after 24 h, the cells were grown in the presence or absence of 25 μm TMZ. Cells were also treated with 100 µm eicosapentaenoic acid (EPA) or docosahexaenoic acid (DHA) (Cayman Chemical, Ann Arbor, MI, USA) complexed with fatty acid-free bovine serum albumin (ALB) (Sigma Aldrich, São Paulo, SP, Brazil) as previously described [27]. ALB was used as the vehicle control (Sigma Aldrich, São Paulo, SP, Brazil). Culture medium and treatments were changed every 24 h. The cells and medium were collected after 72 h and stained with 0.4% trypan blue to distinguish viable from non-viable cells (Sigma Aldrich, São Paulo, SP, Brazil). Cell counts were performed in a Neubauer chamber and all treatments were tested four times in triplicate.

### 4.4. Clonogenic Assay

Control or TMZR cells were seeded in 12-well plates at a concentration of 1 × 10^2^ cells/well, ±25 µm TMZ, in the absence or presence of 100 µm EPA or DHA, and cultured for 7–10 days depending on the cell line. The cells were stained with 0.5% crystal violet and washed to remove excess dye. Images were collected for each well and the number of colonies and the colony area were determined using Image J software (version 1.54p).

### 4.5. Adhesion and Cell Spreading Assay

Control or TMZR cells were cultured in the presence of 100 µm EPA or DHA for 72 h before being collected and seeded in 24-well plates at concentrations of 1 × 10^4^ cells/well in the absence or presence of 25 µm TMZ. As individual cell lines adhered at different rates, the cells were analyzed at 15, 20, 30, 40, 45, or 60 min. At each time point, cells were carefully fixed with 4% formaldehyde in 0.1M phosphate buffer, pH 7.2. The cells were stained with 0.5% crystal violet and washed to remove excess dye. Images were collected from 4 random fields for each well using a Zeiss AxioObserver Z1 inverted microscope (ZEISS Group, Oberkochen, Germany) and the number of adhered cells counted. Cell spreading was measured using Image J software (version 1.54p).

### 4.6. Transwell Migration Assay

The transwell migration assay was performed using a 24-well plate with 8.0 μm pore polycarbonate membrane transwell inserts (Corning), as previously described [71,72]. Initially, cells were seeded onto the top compartment at 2 × 10^4^ cells/well. Cells were kept at 37 °C in a humidified atmosphere with 5% CO_2_ for 2 h until treatment with 100 µm EPA or DHA in the absence or presence of 25 µm TMZ. After 24 h of treatment, the inserts were washed with PBS, and the cells in the upper compartment were removed with a cotton swab. The inserts were then stained with 0.5% crystal violet. The membranes were mounted on glass slides with Entellan (Merck/Millipore, Burlington, MA, USA), and images of 5 random fields per membrane were captured on a Zeiss AxioObserver Z1 inverted microscope and the number of adhered cells were counted.

### 4.7. Lactate and Glucose Assay

Cells were seeded at 3 × 10^4^ cells/well in 24-well plates and after 24 h, the cells were treated with 100 µm EPA or DHA in the absence or presence of 25 µm TMZ. After 72 h, the cells were counted in a Neubauer chamber and the medium was collected to determine lactate and glucose concentration, following previously described methods [11,27,28]. Briefly, glucose was measured using hexokinase to convert glucose to glucose-6-phosphate in the presence of ATP, then glucose-6-phosphate dehydrogenase to convert glucose-6-phosphate to 6-phosphogluconolactone with concomitant conversion of NADP^+^ to NADPH + H^+^. Lactate was measured using lactate dehydrogenase to convert lactate to pyruvate with concomitant conversion of NAD^+^ to NADH + H^+^. Absorbance was measured at 340 nm in an Epoch microplate reader (Biotek, Winooski, VT, USA).

### 4.8. Nile Red Staining of Neutral Lipids

Cells were seeded at 3 × 10^4^ cells/well in 24-well plates on glass coverslips and after 24 h, the cells were treated with 100 µm EPA or DHA complexed with fatty acid-free bovine serum albumin (ALB). ALB was used as the vehicle control. After 72 h, the cells were fixed in 4% formaldehyde freshly prepared from paraformaldehyde, in 0.1 M potassium phosphate, pH 7.2. The cells were stained with 1 µg/mL Nile red and 1 µg/mL DAPI. The coverslips were mounted in Vectashield (Vector Laboratories, Newark, CA, USA) on glass slides and viewed on a Nikon Optiphot-II epifluorescence microscope equipped with a Cool Snap Pro camera and Image Pro Plus software (version 4.1.0.0) as previously described [28,73].

After following the same treatment for 72 h, live cells were stained with Nile red, washed ×3 with PBS, resuspended in PBS and fluorescence measured at λ_ext._ 490 nm and λ_em._ 530 nm using a Synergy H1 Hybrid Multi-Mode Reader (Biotek, Winooski, VT, USA).

### 4.9. Propidium Iodide Staining

Cells were cultured as described in Section 4.8 and after 72 h, live cells were stained with 3 µg/mL propidium iodide. The cells were washed ×3 with PBS, resuspended in PBS, and fluorescence was measured at λ_ext._ 500 nm and λ_em._ 600 nm using a Synergy H1 Hybrid Multi-mode Reader (Biotek, Winooski, VT, USA).

### 4.10. Dihydroethidium Staining

Cells were cultured as described in Section 4.8 and after 72 h, live cells were stained with 1 µg/mL dihydroethidium. The cells were washed ×3 with PBS, resuspended in PBS, and fluorescence was measured at λ_ext._ 500 nm and λ_em._ 600 nm using a Synergy H1 Hybrid Multi-Mode Reader (Biotek, Winooski, VT, USA).

### 4.11. Acridine Orange Staining

Cells were cultured as described in Section 4.8 and after 72 h, live cells were stained with 0.4 µg/mL acridine orange. The cells were washed ×3 with PBS, resuspended in PBS, and fluorescence was measured at λ_ext._ 485 nm and λ_em._ 528 nm and at λ_ext._ 500 nm and λ_em._ 600 nm using a Synergy H1 Hybrid Multi-Mode Reader (Biotek, Winooski, VT, USA).

Cells were cultured as described in Section 4.8 and after 72 h, live cells were stained with acridine orange, mounted on glass slides, and images were captured on a Zeiss AxioObserver Z1 inverted microscope.

Cells were cultured as described in Section 4.8 and after 72 h, live cells were stained with acridine orange then fixed in 4% formaldehyde freshly prepared from paraformaldehyde, in 0.1 M potassium phosphate, pH 7.2. Fixation results in the loss of orange fluorescence emitted by the acridine orange-stained acidic vesicles. Images were captured on a Zeiss AxioObserver Z1 inverted microscope.

### 4.12. DAPI Staining

Cells were cultured as described in Section 4.8 and after 72 h, cells were fixed in 4% formaldehyde freshly prepared from paraformaldehyde, in 0.1 M potassium phosphate, pH 7.2. The cells were stained with 1 µg/mL DAPI in order to visualize nuclear changes associated with apoptosis. The coverslips were mounted in Vectashield (Vector Laboratories, Newark, CA, USA) on glass slides and images were captured on a Zeiss AxioObserver Z1 inverted microscope.

### 4.13. Proteome Profiler Antibody Array

The human phospho-kinase assay was carried out following manufacturer’s instructions (R&D Systems, Minneapolis, MN, USA). After 72 h treatment with EPA or DHA in the presence of TMZ, U87MG TMZR cells were collected and diluted to produce a lysate containing 1 × 10^7^ cells/mL. Briefly, diluted cell lysates were incubated with the nitrocellulose membranes containing capture or control antibody spots overnight. The arrays were washed and incubated with a cocktail of biotinylated detection antibodies. After washing, the membranes were incubated with Streptavidin-HRP, washed again, and finally the membranes were developed with enhanced chemiluminescence (Clarity Western ECL substrate—Bio-Rad Laboratories Inc., São Paulo, Brazil) in a Syngene G-BOX using GeneSys software (version 1.4.3.0). The Image J program was used to determine relative intensities.

### 4.14. Statistical Analysis

All data were plotted and analyzed using GraphPad Prism 10 (GraphPad Prism 10 Software, San Diego, CA, USA). Values express the arithmetic mean ± standard error. Analysis between two groups was performed with Student’s *t*-test. Analysis between three or more groups, considering only one variable, was performed with one-way ANOVA followed by Dunnett’s test. Analysis between three or more groups, considering two variables, were performed with two-way ANOVA followed by a Bonferroni test. The differences were considered statistically significant at *p* < 0.05.

## Data Availability

Access to original data can be provided upon reasonable request.

## Supplementary Materials

The following supporting information can be downloaded at: https://www.mdpi.com/article/10.3390/ijms26188759/s1.

## Abbreviations

The following abbreviations are used in this manuscript:

CNS	Central nervous system
DHA	Docosahexaenoic acid
GLA	Gamma-linolenic acid
EPA	Eicosapentaenoic acid
GBM	Glioblastoma
IDH	Isocitrate dehydrogenase
MDR	Multiple drug resistance
MGMT	O6-methylguanine-DNA methyltransferase
PUFA	Polyunsaturated fatty acids
TMZ	Temozolomide
TMZR	Temozolomide resistant
