# Supplementary material for: The Omega-3 Fatty Acids Eicosapentaenoic Acid and Docosahexaenoic Acid Enhance the Effects of Temozolomide Chemotherapy in Glioblastoma Cells
Source: Int J Mol Sci. 2025 Sep 9;26(18):8759. doi: 10.3390/ijms26188759 (PMC12469626; doi:10.3390/ijms26188759)
Supplement: Supplementary file 1 [file ijms-26-08759-s001.zip › ijms-3769858-supplementary.pdf]

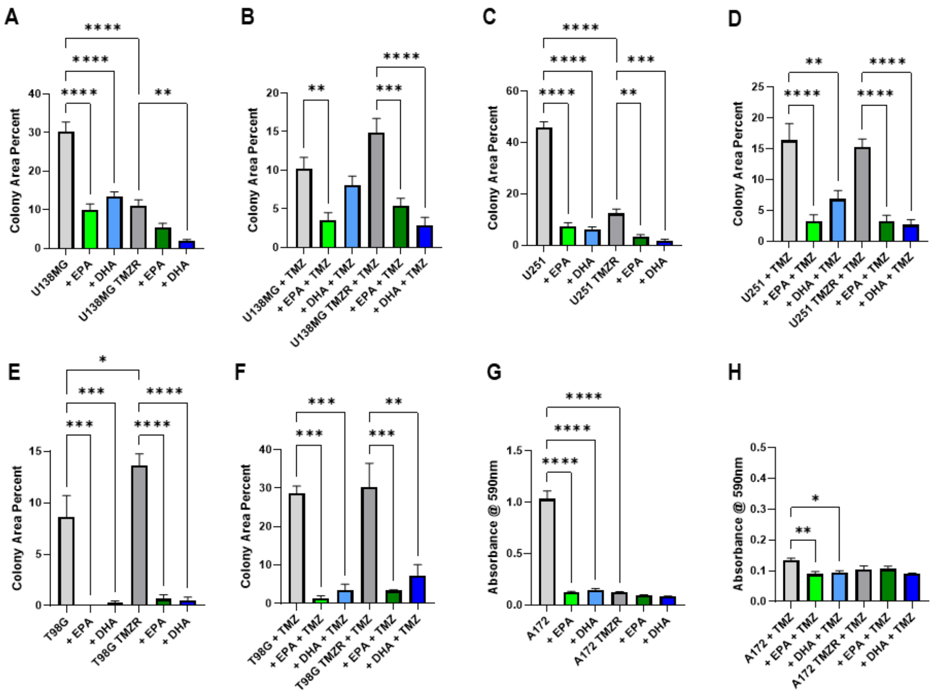

Supplementary Figure S1. - Effect of 100μM EPA or DHA in the absence or presence of 25μM temozolomide on clonogenic capacity of control or TMZ-resistant cells. Colony area percentages were analyzed using Image J software, absorbance at 590nm was quantified by spectrophotometry. Data are presented as mean ± SEM, N = 3-4. Differences were considered significant at $p < 0.05$. * = $p < 0.05$; ** = $p < 0.01$; *** = $p < 0.001$; ****= $p < 0.0001$.

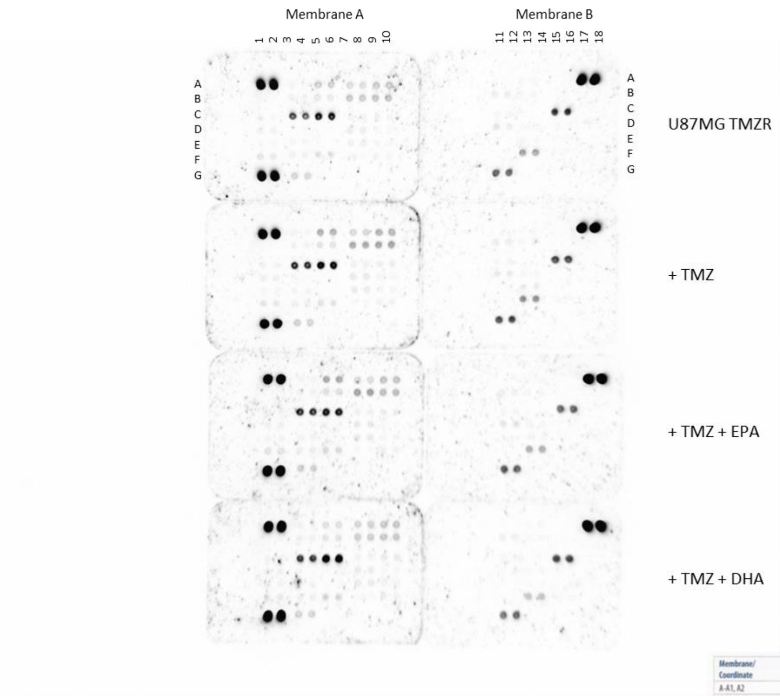

Membrane/ Coordinate	Target/Control	Phosphorylation Site	DuoSet® IC (Total) ELISA Development System	DuoSet® IC (Phospho) ELISA Development System
A-A1, A2	Reference Spot	——	——	——
A-A3, A4	p38α	T180/Y182	DYC8691B	DYC869B
A-A5, A6	ERK1/2	T202/Y204, T185/ Y187	——	DYC1018B
A-A7, A8	JNK 1/2/3	T183/Y185, T221/ Y223	DYC1205	DYC1387
A-A9, A10	GSK-3α/β	S21/S9	DYC2157	DYC2630
B-A13, A14	p53	S392	DYC1043	DYC2996
B-A17, A18	Reference Spot	——	——	——
A-B3, B4	EGF R	Y1086	DYC1854	——
A-B5, B6	MSK1/2	S376/S360	——	——
A-B7, B8	AMPKα1	T183	DYC3197	DYC3528
A-B9, B10	Akt 1/2/3	S473	——	DYC887B
B-B11, B12	Akt 1/2/3	T308	——	——
B-B13, B14	p53	S46	DYC1043	DYC1489
A-C1, C2	TOR	S2448	——	DYC1665
A-C3, C4	CREB	S133	——	DYC2510
A-C5, C6	HSP27	S78/S82	DYC1580	DYC2314
A-C7, C8	AMPKα2	T172	——	——
A-C9, C10	β-Catenin	——	DYC1329	——
B-C11, C12	p70 S6 Kinase	T389	DYC8962	DYC896
B-C13, C14	p53	S15	DYC1043	DYC1839
B-C15, C16	c-Jun	S63	——	——
A-D1, D2	Src	Y419	——	DYC2685
A-D3, D4	Lyn	Y397	——	DYC3936
A-D5, D6	Lck	Y394	——	——
A-D7, D8	STAT2	Y689	——	——
A-D9, D10	STAT5a	Y694	——	——
B-D11, D12	p70 S6 Kinase	T421/S424	DYC8962	DYC8965
B-D13, D14	RSK1/2/3	S380/S386/S377	——	DYC889B
B-D15, D16	eNOS	S1177	——	——
A-E1, E2	Fyn	Y420	——	——
A-E3, E4	Yes	Y426	——	DYC3929
A-E5, E6	Fgr	Y412	——	——
A-E7, E8	STAT6	Y641	——	——
A-E9, E10	STAT5b	Y699	——	——
B-E11, E12	STAT3	Y705	——	DYC4607B
B-E13, E14	p27	T198	DYC2256	——
B-E15, E16	PLC-γ1	Y783	——	——
A-F1, F2	Hck	Y411	——	——
A-F3, F4	Chk-2	T68	——	DYC1626
A-F5, F6	FAK	Y397	DYC4467	DYC4528
A-F7, F8	PDGF Rβ	Y751	DYC385	DYC3096
A-F9, F10	STAT5a/b	Y694/Y699	——	——
B-F11, F12	STAT3	S727	——	——
B-F13, F14	WNK1	T60	——	DYC4720
B-F15, F16	PYK2	Y402	——	——
A-G1, G2	Reference Spot	——	——	——
A-G3, G4	PRAS40	T246	——	DYC6890
A-G9, G10	PBS (Negative Control)	——	——	——
B-G11, G12	HSP60	——	DYC1800	——
B-G17, G18	PBS (Negative Control)	——	——	——

Supplementary Figure S2. – Effect of 100μM EPA or DHA on phosphoprotein profile of U87MG TMZR cells in the presence of 25μM temozolomide. Array layout, target proteins and phosphorylation sites.
